# Low Glucose Concentrations Induce a Similar Inflammatory Response in Monocytes from Type 2 Diabetic Patients and Healthy Subjects

**DOI:** 10.1155/2017/9185272

**Published:** 2017-10-31

**Authors:** Francesco Piarulli, Giovanni Sartore, Annalisa Sechi, Daniela Basso, Paola Fogar, Eliana Greco, Eugenio Ragazzi, Annunziata Lapolla

**Affiliations:** ^1^Department of Medicine (DIMED), University of Padova, Padova, Italy; ^2^Regional Coordination Centre for Rare Diseases, University Hospital of Udine, Udine, Italy; ^3^Department of Pharmaceutical and Pharmacological Sciences (DSF), University of Padova, Padova, Italy

## Abstract

This study aims to assess the proinflammatory interleukin 1*β* (IL-1*β*) and anti-inflammatory IL-10 production by monocytes from 38 patients with type 2 diabetes and 31 controls in different glucose concentrations. Monocytes were incubated in low (2.5 mmol/L)-, normal (5.0 mmol/L)-, and high (20 mmol/L)-glucose conditions in the presence and absence of lipopolysaccharide (LPS). Monocytes from both patients and controls only produced a significant increase in IL-1*β* in low-glucose conditions (*p* < 0.01), and this phenomenon was amplified in the presence of LPS, while it was not seen in normal- or high-glucose conditions, not even in the presence of LPS stimulation. There was no increase in IL-10 production by monocytes from either diabetic patients or controls using whatever glucose concentrations, except when treated with LPS in normal-glucose conditions. These findings seem to suggest that low-glucose conditions induce an inflammatory response in monocytes in all individuals, as an intrinsic capacity of this cell line. On the other hand, monocytes only retain their anti-inflammatory ability in response to known inflammatory stimuli such as LPS, under normal-glucose concentrations. In conclusion, human monocytes express an inflammatory pattern in low-glucose conditions *in vitro*. This response could contribute to explaining the higher cardiovascular risk induced by hypoglycemia in diabetic patients.

## 1. Introduction

Diabetes is associated with accelerated atherosclerosis and a much increased risk of cardiovascular disease. Hyperglycemia and oxidative stress lead to tissue damage, thus contributing to cardiovascular complications due to the activation of cellular inflammatory and proliferative processes, in which circulating monocytes have a crucial role [1, 2]. Several studies have demonstrated that monocytes become more prone to inflammation in conditions involving high glucose levels. *In vitro*, human monocytes are activated by high glucose concentrations and prompted to express key proinflammatory cytokines such as interleukin 1*β* (IL-1*β*), which is more strongly associated with inflammation than any other members of the IL-1 family [3, 4]. Circulating levels of IL-1 are associated with traditional cardiac risk factors, such as diabetes mellitus, hypertension, smoking, and dyslipidemia. Elevated levels of IL-1 result in an inflammatory cascade that promotes atherosclerosis and plaque destabilization [5].

On the other hand, mononuclear cells have powerful anti-inflammatory properties thanks to the production of IL-10 [6, 7]. It has been demonstrated that patients with type 2 diabetes have a reduced capacity for IL-10 production [8, 9].

Another important aspect to consider in the vascular complications of diabetes is the role of hypoglycemia. *In vivo*, low glucose concentrations cause profound changes in the cardiovascular system and several hematological parameters, such as platelet activation and leukocytosis [10, 11]. Nematollahi et al. found that hypoglycemia coincides with higher indexes of inflammation and oxidative stress in healthy subjects [12]. Hypoglycemic conditions induce an upregulation in the membrane's expression of monocyte glucose carriers (GLUT 3 in particular) with a consequently greater immune and inflammatory cell activation [13, 14]. More recently, two studies have shown that hypoglycemia induces proinflammatory changes in type 1 diabetic patients too, including an increase in the levels of proinflammatory mediators [15, 16].

Conflicting data are available on how monocytes respond to low glucose levels in type 2 diabetes, and it is not clear whether monocyte activation differs between healthy subjects and patients with type 2 diabetes in low-glucose conditions. The purpose of this study was to contribute to clarifying these issues.

## 2. Material and Methods

### 2.1. Material

We studied a sample of 69 subjects: 38 patients with type 2 diabetes mellitus and 31 age-matched healthy subjects enrolled as a control group. A history of chronic or recent acute inflammatory disease was ruled out in all subjects involved in the study. The patients had been diabetic for at least 5 years. Glycemic control was assessed by testing HbA1c levels.

Ninety per cent of the patients were taking oral glucose-lowering drugs, while none were on insulin therapy. Ten patients were taking statins, 26 were being treated with common antihypertensive drugs, and 9 were taking aspirin 100 mg/day. None of the healthy controls had recently taken any medication.

The study described in this paper was conducted in accordance with the Helsinki Declaration (2000) of the World Medical Association and approved by the local ethics committee. All participants gave their written informed consent.

A blood sample was obtained from each participant after an overnight fast on the day of the study. HbA1c was assayed using high-performance liquid chromatography (Bio-Rad, Milan, Italy) [17]. Plasma glucose was measured using a glucose oxidase method [18].

### 2.2. Measurement

The aliquot of blood (20 mL) collected with EDTA K3 was processed within 2 hours of sampling to isolate CD14^+^ monocytes, which were purified in two steps: first by negative selection using a RosetteSep kit (StemCell Technologies, Voden Medical Instruments Spa, Milano, Italy) and then by Ficoll gradient centrifugation (Histopaque®-1077, Sigma-Aldrich, Milano, Italy). The recovery rate measured by flow cytometry was in the range of 60–90%.

The purified monocytes were counted in Burker's chamber and resuspended in culture medium (RPMI supplemented with 0.1% gentamicin, 10% FCS, and 1% glutamine; Invitrogen, San Giuliano Milanese, Italy) at a concentration of 1 × 10 cells/100 *μ*L before the experimental procedure.

Monocytes were aliquoted in three tubes at a density of 8 × 10^5^ cells/tube, centrifuged at 2000*g*, and washed once in PBS buffer. The cells were then resuspended in 800 *μ*L of Dulbecco's glucose-free modified Eagle medium supplemented with 0.1% gentamicin, 10% FCS, and 2% glutamine, with three different glucose concentrations: (i) low (2.5 mmol/L), (ii) physiological (5 mmol/L), and (iii) high (20 mmol/L). Each of the three aliquots of monocytes was split into two separate wells in a 24-well plate to obtain a final density of 400 × 10^3^ monocytes/well (1.5 cm^2^ growth area). They were cultured at 37°C with 5% CO_2_. Twenty hours after seeding, the previously split monocyte samples were left either untreated or treated with 1 *μ*g/mL lipopolysaccharide (LPS, Sigma-Aldrich GmbH, Germany). LPS is believed to be one of the most potent activators of monocytes in order to increase cytokine production [19]. After another 4 hours, the supernatants were collected, centrifuged at 2000*g* to remove floating cells or cell debris, and then stored at −80°C until the cytokine content was measured.

The following cytokines were measured in the supernatants of monocytes using enzyme-linked immunosorbent assays (ELISAs) according to the manufacturer's instructions: IL-1*β* (CV = 6.4%) (BioSource, Nivelles, Belgium) and IL-10 (CV = 9%) (Medical System, Genova, Italy).

### 2.3. Statistical Analysis

Data are expressed as mean values ± SD or SEM, as stated in the Results. Statistically significant differences between several groups were assessed by ANOVA. Within-subject changes under different experimental conditions were determined using Student's *t*-test for paired data. A *p* value < 0.05 was considered statistically significant.

## 3. Results

The main clinical parameters in the controls and diabetic patients are shown in Table 1.

The diabetic patients had significantly (*p* < 0.01) higher fasting plasma glucose, hemoglobin A1c (HbA1c), and systolic and diastolic blood pressure values.

**Figure 1:**
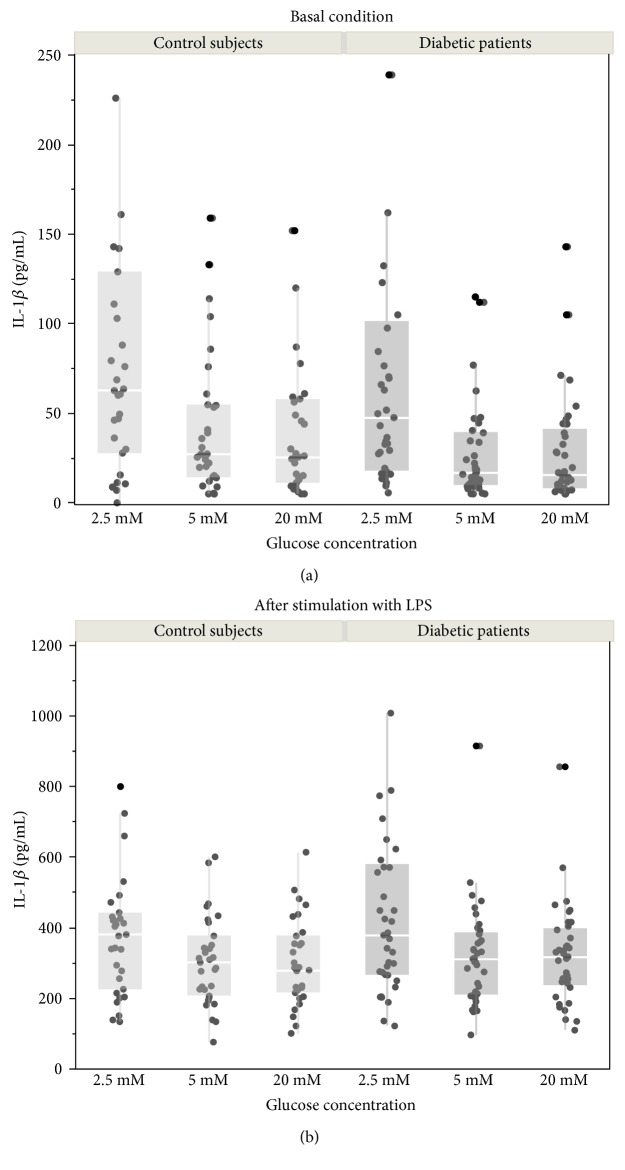
IL-1*β* released by monocytes obtained from diabetic patients (*n* = 38) and control subjects (*n* = 31). (a) Basal conditions. (b) After stimulation with LPS (1 *μ*g/mL). Dot plots show individual absolute values measured in the culture medium at different glucose concentrations: glucose 2.5 mmol/L, 5.0 mmol/L, and 20 mmol/L. Graphs are completed with box plots, where the ends of the box represent the 25th and 75th quantiles, respectively, while the horizontal line within the box represents the median value; whiskers extend the distances computed as 1st quartile − 1.5 × interquartile range and 3rd quartile + 1.5 × interquartile range. Following a significant two-way ANOVA, comparisons between glucose concentrations have been evaluated as paired data (see Table 2 for detailed statistics). No significant differences were detected with one-way ANOVA between controls and diabetic patients at each glucose concentration.

A statistical analysis was conducted by means of a paired comparison between the monocytes obtained from the same individual (healthy control or diabetic patient) under different culture conditions. An overall view of data distribution is presented in Figure 1. In order to increase the statistical power and reduce the effects of possible confounders, comparisons between different glucose concentrations were obtained considering paired data from the same subject.  Table 2 shows the paired differences for the IL-1*β* (part (a)) and IL-10 (part (b)) concentrations by glucose level in the culture medium, with (LPS^+^) and without (LPS^−^) stimulation. In these paired comparisons, a negative value indicates a higher cytokine concentration in the latter condition.

Table 2(a) shows the comparison between normal (5 mmol/L)- and low (2.5 mmol/L)-glucose conditions: incubation at low glucose concentrations coincided with a significant increase in IL-1*β* production by the monocytes cultured from both controls (with [*p* < 0.01] or without LPS [*p* < 0.01]) and diabetic patients (with [*p* < 0.01] or without LPS [*p* < 0.05]). The comparison between high (20 mmol/L)- and normal (5 mmol/L)-glucose conditions showed no significant differences in the IL-1*β* levels measured in the monocyte cultures from either group (controls or diabetic patients), before or after LPS stimulation.

In Table 2(b), the comparison between normal (5 mmol/L)- and low (2.5 mmol/L)-glucose conditions showed no significant differences in IL-10 levels in the monocyte cultures from either controls or diabetic patients. The comparison between high (20 mmol/L)- and normal (5 mmol/L)-glucose conditions, without any LPS stimulation, showed no significant differences in IL-10 levels in the monocyte cultures from either group, but after stimulation with LPS, the IL-10 levels increased significantly (*p* < 0.01) under normal-glucose conditions in both groups.

No statistically significant differences emerged on one-way ANOVA in the mean concentrations of IL-1*β* and IL-10 released by the monocytes cultured under the different experimental conditions (low, normal, and high glucose concentrations, with and without LPS stimulation) between the control group and the diabetic group (Figure 1).

## 4. Discussion

The present study suggests that low glucose concentrations (but not normal and high glucose levels) can affect the *in vitro* inflammatory response of human monocytes. In our experiment, low-glucose conditions prompted a greater production of the proinflammatory cytokine IL-1*β* by the monocytes from both type 2 diabetic patients and healthy controls. This evidence emerged when we compared the differences between the mean concentrations of IL-1*β* released by the monocytes cultured with three different glucose concentrations (low, normal, and high), before and especially after stimulation with LPS, which is known to stimulate monocyte inflammatory response [20]. It has been reported that hypoglycemic conditions induce an upregulation of the glucose transporter GLUT 3 in the monocyte's plasma membrane [13] and that LPS amplifies GLUT 3 overexpression [21].

GLUT 3 upregulation represents an autoregulatory mechanism to ensure an adequate glucose supply to the cells and thus protect leukocytes against the detrimental effects of low glucose levels, triggering an inflammatory cell activation as well. Nematollahi et al. demonstrated that hypoglycemia induced by a standard insulin tolerance test caused an increase in proinflammatory cytokines (TNF*α*, IL-1*β*, IL-6, and IL-8) and oxidative stress in healthy individuals [12]. Hypoglycemia can prompt an acute increase in inflammatory and proatherothrombotic biomarkers in patients with type 1 diabetes and healthy individuals [15]. Wright et al. also showed that acute hypoglycemia induced by hyperinsulinemic clamping led to increased CD40 expression (an index of inflammatory activation) on monocytes in type 1 diabetic patients [16].

In this context, our study supports the part played by monocyte activation in the inflammatory state induced by low glucose levels, both in patients with type 2 diabetes and in healthy individuals. The above-mentioned studies were all based on the induction of hypoglycemia *in vivo* by means of insulin infusions, however, whereas the low glucose levels in our study were obtained *in vitro*, in the absence of insulin or any counter-regulatory hormone activation, so any interference can be ruled out. Hypoglycemia *in vivo* can indeed induce an increase in hormonal adrenergic activity, which gives rise to a greater inflammatory stress [22].

Unlike other studies, but in agreement with Gonzalez et al. [23], our results indicate that IL-1*β* production was unaffected by high glucose concentrations in either type 2 diabetic patients or healthy controls. These inconsistent results could be due to differences in the experimental conditions adopted [24], such as the glucose concentrations or incubation times used before measuring the cytokine levels [4, 6, 23]. The data generated seem to suggest that low-glucose conditions induce an inflammatory response of monocytes in all individuals, as an intrinsic capacity of this cellular line.

Monocytes only retain their anti-inflammatory ability (represented by IL-10 production) in response to a known inflammatory stimulus (LPS) in normal-glucose conditions. A similar picture has been seen *in vivo* [9], when type 2 diabetic patients' metabolic control was fairly normal. This potentially protective mechanism seems to be disrupted when glucose concentrations are altered.

On another point of view, a possible future area of investigation may also consider the epigenetic aspect of monocyte modulation in dysglycemic situations. It has been reported that high glucose levels induce proinflammatory cytokines via epigenetic changes [25, 26]. Identification of molecular links to monocyte proinflammatory cytokines via transcription mechanisms and nuclear chromatin remodeling may lead to possible therapeutic targets that, besides influencing inflammatory processes, can also improve glucose metabolism. Promising natural compounds have already been identified, such as curcumin [25].

To our knowledge, this is the first study to provide evidence that *low glucose* concentrations may be a powerful stimulus for proinflammatory cytokine expression by human monocytes. The proinflammatory and proatherogenic changes induced in monocytes by low *blood* glucose concentrations may be partly responsible for the increase in diabetic patients' cardiovascular risk with higher rates of hypoglycemia, as identified in the ACCORD study [27]. These findings support the need to prevent hypoglycemic conditions to reduce CV events.

## Figures and Tables

**Table 1:** Clinical parameters for the healthy controls and type 2 diabetic patients.

	Controls (*n* = 31)	Type 2 diabetic patients (*n* = 38)	*p*
Age (years)	61.9 ± 11.3	61.2 ± 6.8	NS
Duration of disease (years)	—	11.8 ± 3.9	—
FPG (mg/dL)	98.8 ± 10.4	168.9 ± 37.2	<0.001
HbA1c (%)	5.45 ± 0.31	7.40 ± 1.05	<0.001
DBP (mmHg)	76 ± 8	83 ± 8	<0.001
SBP (mmHg)	128 ± 10	137 ± 15	<0.001

Data are expressed as mean ± SD. *p* values were calculated using Student's *t*-test for unpaired data. FPG: fasting plasma glucose; HbA1c: hemoglobin A1c; DBP: diastolic blood pressure; SBP: systolic blood pressure.

**(a):** 

IL-1*β* paired differences (pg/mL)	Controls (*n* = 31)	Controls (*n* = 31)	Diabetic patients (*n* = 38)	Diabetic patients (*n* = 38)
	LPS^−^	LPS^+^	LPS^−^	LPS^+^
G5 − G2.5	−56 ± 17^∗∗^	−94 ± 28^∗∗^	−79 ± 32^∗^	−171 ± 57^∗∗^
G20 − G5	3 ± 8	−6 ± 10	−8 ± 8	0.5 ± 9.6

**(b):** 

IL-10 paired differences (pg/mL)	Controls (*n* = 31)	Controls (*n* = 31)	Diabetic patients (*n* = 38)	Diabetic patients (*n* = 38)
	LPS^−^	LPS^+^	LPS^−^	LPS^+^
G5 − G2.5	−3 ± 1	−5 ± 6	−3 ± 2	29 ± 20
G20 − G5	0 ± 1	−21 ± 4^∗∗^	−2 ± 1	−36 ± 9^∗∗^

G2.5: glucose 2.5 mmol/L; G5: glucose 5.0 mmol/L; G20: glucose 20 mmol/L. Data are mean ± SEM. Paired comparisons were obtained by changing one experimental variable at a time. Negative values indicate a higher IL-1*β* (a) and IL-10 (b) concentration in the latter condition considered. ^∗^*p* < 0.05, ^∗∗^*p* < 0.01. Student's *t*-test for paired data indicating a significant change between the paired experimental conditions stated in the first column.
